# Atenção primária à saúde no Brasil: uma história tecida em pensamento e práticas

**DOI:** 10.1590/S0104-59702023000100058

**Published:** 2023-10-23

**Authors:** Márcia Valéria Morosini

**Affiliations:** i Escola Politécnica de Saúde Joaquim Venâncio Fiocruz Rio de Janeiro RJ Brasil marcia.morosini@fiocruz.br Professora e pesquisadora,Escola Politécnica de Saúde Joaquim Venâncio/Fiocruz.Rio de Janeiro – RJ – Brasil marcia.morosini@fiocruz.br

A atenção primária à saúde (APS) no Brasil encontra-se num momento decisivo. Tendo sobrevivido a quatro anos de gestão deletéria do Sistema Único de Saúde (SUS), orientada pela racionalidade do mercado, favorecedora dos interesses do setor privado em detrimento dos direitos sociais, a APS avista um horizonte de possibilidades no início do novo governo federal. É tempo de realizar o balanço das perdas, a avaliação do que precisa ser modificado ou preservado e definir a direção que a APS deve seguir daqui por diante. Nessa oportunidade retornam, parcialmente modificados e atualizados, interesses e concepções distintos, muitas vezes opostos que se fizeram presentes repetidamente nos processos de disputa pela conformação do que costumamos denominar nacionalmente atenção básica.

Neste momento de transição na política nacional, faz-se particularmente oportuna a temática do livro *Atenção primária à saúde: uma história brasileira* (Paiva, Pires-Alves, 2021), que revisita a história da APS como campo de pensamento e prática, colocando em foco as propostas e experiências que marcaram conjunturas importantes para sua estruturação no Brasil. Seus autores, Carlos Henrique Paiva e Fernando Pires-Alves, peritos no ofício de historiador, ao voltar ao passado partem de uma premissa importante: não procurar aspectos de continuidade linear entre ideias e modos de organização ao longo do tempo, e sim identificar o que chamam de conexões, que lhes permitem compor uma “história comum” da APS, ainda que tecida por elementos diversos e contraditórios.

Nesse processo, os autores optam por uma perspectiva ampliada, abrangendo os experimentos de expansão dos serviços básicos de saúde anteriores à realização da Conferência de Alma-Ata. Desse modo, a trajetória de formulações relativas à APS se alonga, tornando-se também mais diversificada, e Alma-Ata se desloca de lugar originário para marco de referência e sistematização de propostas para o campo, que passam a se difundir mundialmente.

Assumem a condição polissêmica da APS e enfrentam o desafio de compreender a dinâmica conflituada de um campo sobre o qual não cabe falar exatamente em recuos e avanços, mas em movimentos contraditórios resultantes da interação entre política, ciência e luta social. Correspondem a posições técnicas e políticas que competem pelo delineamento da APS não somente no Brasil, mas em diferentes realidades. Incluem a definição da abrangência populacional, do escopo e conteúdo das práticas e dos serviços ofertados, da força de trabalho, da tecnologia e dos diversos recursos empregados, da responsabilidade pelo provimento da atenção, delimitando o espaço entre o público e o privado. Os resultados dessas disputas dependem da correlação das forças sociais nos diversos contextos. No Brasil, seus resultados variam no tempo e se apresentam frequentemente de modo combinado.

O livro contempla cinco conjunturas diferentes e, até certo ponto, disruptivas sobre as quais os autores se debruçaram, pesquisando acervos de documentos nacionais e internacionais, assim como a literatura do campo. As descrições e análises desenvolvidas sobre esses períodos abordam dimensões da política, da organização administrativa, da estruturação dos serviços e das concepções e práticas vigentes, compondo os cinco capítulos da obra. Cada capítulo é encerrado com a apresentação de cópia, na íntegra, de um documento da época, ilustrativo das ideias discutidas ou de algum ponto de destaque.

A tessitura das discussões abrange a compreensão do contexto internacional, suas influências na realidade brasileira e pontos de encontro entre os interesses externos e internos ao país. É destacada a atuação dos organismos internacionais, como a Organização Mundial de Saúde e a Organização Pan-americana da Saúde, como também de instituições da sociedade civil, como a Fundação Rockefeller, que protagonizou a promoção do modelo dos centros de saúde nos EUA e nos países sob sua influência político-econômica. As mudanças de enfoque, de posição e de direcionamento nessas instituições acompanham mudanças nas políticas mundiais e locais. Os poderes locais, ainda que subalternizados, têm seu papel demarcado no delineamento das políticas nacionais.

É assim, nesse vai e vem entre o plano internacional e o nacional, que a obra percorre a história da APS. Nos diferentes contextos são identificadas e analisadas as formas de organizar os serviços, o cuidado, o trabalho em saúde, o aparato tecnológico empregado, a aplicação dos recursos disponíveis, incluídas a força de trabalho e sua formação. Localizando debates e políticas estruturantes do campo da saúde pública e seus desenvolvimentos ulteriores, os autores percebem a formação do pensamento que toma como objeto particular as questões organizacionais da saúde e as modificações e tensões que sua matriz percorre. Iluminam também o processo de configuração do campo profissional do sanitarista brasileiro cuja constituição se associa de modo importante à história da APS.

O terreno comum no qual se assentam as experiências da APS é feito de ideias que se consolidam em seu percurso histórico, alcançando o presente: hierarquização e funcionamento em rede dos serviços de saúde; regionalização; territorialização do cuidado; treinamento de pessoal leigo para atuar nos cuidados básicos, especialmente na educação em saúde, entre outros. Manifestam-se em propostas do Movimento da Reforma Sanitária, na construção dos princípios e diretrizes do SUS e na configuração da política nacional para a APS no Brasil: a Política Nacional de Atenção Básica (Pnab) (Brasil, 2006).

A obra termina nesse momento instituinte da primeira Pnab e nos coloca diante da potência de uma política em construção, visando à extensão do direito à saúde, numa conjuntura adversa, de hegemonia do ideário neoliberal, cujos limites foram tensionados e estendidos de modo que se logrou conceber e difundir a Estratégia Saúde da Família como modelo de reorientação da atenção à saúde.

Voltando ao presente, encontramos os desafios colocados pela atual Pnab (Brasil, 2017) que pavimenta o caminho para a ampliação da privatização da APS e enfraquece os atributos que a tornam uma política pública orientada para a universalidade do direito e a integralidade do cuidado. Na retomada do processo de consolidação do SUS, a APS tem papel fundamental e precisa recuperar e fortalecer sua capacidade inovadora e estruturante do sistema de saúde, articulado e integrado a partir de uma base territorializada, com orientação comunitária e cuidado multiprofissional, contínuo, realizado num processo de trabalho mais horizontalizado e participativo.
